# A Comprehensive Quantitative Assessment of Bird Extinction Risk in Brazil

**DOI:** 10.1371/journal.pone.0072283

**Published:** 2013-08-12

**Authors:** Nathália Machado, Rafael Dias Loyola

**Affiliations:** 1 Conservation Biogeography Lab, Departamento de Ecologia, Universidade Federal de Goiás, Goiânia, Goiás, Brazil; 2 Programa de Pós-Graduação em Ecologia e Evolução, Universidade Federal de Goiás, Goiânia, Goiás, Brazil; University of Kent, United Kingdom

## Abstract

In an effort to avoid species loss, scientists have focused their efforts on the mechanisms making some species more prone to extinction than others. However, species show different responses to threats given their evolutionary history, behavior, and intrinsic biological features. We used bird biological features and external threats to (1) understand the multiple pathways driving Brazilian bird species to extinction, (2) to investigate if and how extinction risk is geographically structured, and (3) to quantify how much diversity is currently represented inside protected areas. We modeled the extinction risk of 1557 birds using classification trees and evaluated the relative contribution of each biological feature and external threat in predicting extinction risk. We also quantified the proportion of species and their geographic range currently protected by the network of Brazilian protected areas. The optimal classification tree showed different pathways to bird extinction. Habitat conversion was the most important predictor driving extinction risk though other variables, such as geographic range size, type of habitat, hunting or trapping and trophic guild, were also relevant in our models. Species under higher extinction risk were concentrated mainly in the Cerrado Biodiversity Hotspot and were not quite represented inside protected areas, neither in richness nor range. Predictive models could assist conservation actions, and this study could contribute by highlighting the importance of natural history and ecology in these actions.

## Introduction

The current biodiversity crisis has stimulated a rising tide of questions about species extinction, most of them focusing on the mechanisms making some species more prone to extinction than others [1]–[4]. Previous studies have reported several threats that are directly associated with species extinction, including habitat loss and fragmentation, hunting, climate change, and alien species invasion [5]–[8]. However, not all species respond equally to these threats as a result of evolutionary history, behavior, and intrinsic biological features [9], [10]. This serves to explain why, apart from a plethora of external threats, the major components of extinction risk relate to intrinsic features of a particular species [2], [11],some features being positively or negatively associated with extinction risk. This is the case with body mass [11]–[15], size of geographic range [2], [11], [16], [17], clutch size [1], fecundity [13], and diet [3], [14]. Therefore, extinction pathways are usually defined by an inter independent combinations of these multiple features [2], [3], [15], [16], [18]. In this study, we modeled extinction risk based on several biological features and external threats, and showed to what extent these variables define multiple species-specific pathways to extinction, which are also known to be geographically structured.

Extinction risk models produce robust quantitative predictions and can help to evaluate how extinction risk is geographically structured, a crucial step in developing effective conservation actions aimed at reducing species loss [19]–[21]. Yet, our knowledge on how multiple factors interact to define extinction risk is still limited, even for well-known vertebrates such as birds [22]. In fact, several studies have established the significance of birds as mobile links in the dynamics of natural and human-dominated ecosystems [23], [24]. They also serve as good indicator groups for establishing conservation actions [25], even when aiming at the surrogacy of functional and phylogenetic diversity [26].

Although Brazil harbors some of the richest avian fauna in the world, the country also contains the largest number of critically endangered bird species [27]. Our goal was to use the birds of Brazil as a case study to quantify and map extinction risk based on the biological features of species and external threats. We also evaluated how the current level of protected land areas in Brazil relates to such risk. In particular, we want to (a) understand how specific variables set pathways to bird extinction, (b) define which variables contribute most in defining extinction risk and the thresholds between threatened and non-threatened species, (c) evaluate the geographic structure of extinction risk in Brazil, and (d) quantify how much of the geographic range of species predicted to be under high and low extinction risk is actually contained by the current network of protected areas in Brazil.

## Methods

### Data

We compiled a database of bird species occurring in Brazil along with their respective biological features (n = 1557 species, excluding marine birds) and identified external threats. We chose biological features and external threats based on their role in defining extinction risk in the literature as well as on the basis of data availability. We used the following features: (1) total geographic range size in km^2^ (based on digital maps provided by the BirdLife International [28], available at http://www.birdlife.org/datazone/info/spcdownload), (2) mean body mass in grams (not considering intraspecific variation or sexual dimorphism), (3) trophic guild (nectarivore, frugivore, piscivore, herbivore, carnivore, detritivore, insectivore, granivore and omnivore), (4) diet breadth (the total number of items in their diet), (5) type of habitat (the preferred habitat that the particular species inhabits: freshwater, forest, shrub, and grassland), (6) habitat breadth (number of habitats which the species occupied), (7) clutch size, (8) elevational amplitude distribution (in meters), (9) activity period (diurnal, nocturnal or both), and (10) migratory behavior (non-migrants or migrants). We compiled these data from several different sources (see Appendix A).

We also compiled external threats for each species from the Red List of Threatened Species published by the International Union for Conservation of Nature (IUCN) [29]. These threats were: (11) habitat conversion, (12) hunting or trapping, (13) diseases, (14) climate change and (15) invasive species. We associated external threats to each species according to the information available in the IUCN Red List. These threats were treated as a binary variable in our analysis. For instance, if a given species was threatened by climate change according to IUCN, it was assigned a "1" in the data matrix. Otherwise, if the species was not threatened by climate change, then it received a value of "0" (zero). We repeated the process for all external threats listed above. Therefore, more than a single factor could threaten a species, but each threat was treated independently. This methodology allowed us to include external threats in our analysis, while retaining all the statistical properties and power of using decision trees (see below).

We also used the threat status of all species according to the IUCN Red List [29], which assigns species to different threat categories according to distinct criteria: Criterion A – species threatened because of a recent decline in population, Criterion B – species threatened because of a limited geographic range, Criteria C and D – species threatened because of low abundance (<2500 individuals, and <250 individuals, respectively) and Criteria E – species threatened based on quantitative analysis. We did not include species listed solely under criterion B (n = 17) to avoid potential circularity in extinction risk models given that we included range size as a predictor of extinction risk in our analyses. Also, there were no species listed under criterion E in Brazil. We considered any species classified as Least Concern (LC) and Near Threatened (NT) as ‘non-threatened species’, whereas those species classified as Vulnerable (VU), Endangered (EN), and Critically Endangered (CR) were classified ‘threatened species’ following the IUCN [28].

We downloaded data detailing the network of protected areas in Brazil (PAs) from the IUCN and UNEP-WCMC [30]. In our analyses, we considered those areas classified I-IV by the IUCN as ‘strictly protected areas’ and those areas classified VI as ‘not-strictly protected areas’ [31].

### Analyses

We modeled extinction risk using decision tree, a logical model represented by a tree that shows how the response variable (in our case, threat status) could be predicted by explanatory variables (here, bird biological features and external threats) [32]. When the decision tree has a categorical response (such as our response: threatened or non-threatened) it is called a classification tree. This technique was designed to deal with complex interactions, such as those related to extinction risk, which can differ among taxa in non-linear ways, and should not be viewed as hypothesis-testing routines, instead as hypothesis-generating [32]–[34]. Because this method is capable of dealing with complex interactions among variables, it has been applied for a number of ecological data analyses. In our case, decision tree models are particularly useful as they do not assume/require any specific statistical distribution for the predictor variable or data independence, avoiding potential concerns about pseudo-replication [22], [34]. The main issue, when not controlling for the phylogenetic structure of extinction risk, is that species cannot be considered statistically independent, owing to phylogenetic autocorrelation. This autocorrelation can generate spurious values for degrees of freedom and p-values, biasing the results. However, this is not an issue in decision tree models because they do not test for significance, therefore any level of inflation in degrees of freedom does not alter the structure of trees [33]. Furthermore, our models did not attempt to unravel the evolutionary basis of bird extinction risk. Given that we were not using a linear regression model, there was no need to consider the phylogenetic relationship between species and associated practical problems of generating independence (Please see Jones & Sullivan [22], for more details).

The model splits the initial dataset into homogeneous subsets in terms of the response variable, using a single predictor variable at each node. We measured homogeneity within subsets using the Gini Index [32]. The initial result of a classification tree is usually a large tree that could be over-fitted. Therefore, we pruned the tree to its optimal size using results from 10 cross-validations, establishing a trade-off between prediction accuracy and model complexity [32]. Then, to estimate a proportion of extinction risk we divided the number of threatened species (according to IUCN Red List) by the total species at each node.

To evaluate the contribution of each variable in predicting extinction risk, we built 499 random classification trees using random forest. Random forest is a method that builds several independent classification tree models (excluding each predictor variable from the model at each time) and combines the results of all trees [32]. By comparing the accuracy of the models built by random forest with those built by our original classification tree, we could assess the importance of each variable used to predict extinction risk.

To evaluate model accuracy we used the Cohen’s Kappa statistics (package ‘irr’ in the R software [35]) to measure the concordance between the species categorization generated by the model and their current status based on the IUCN Red list (threatened or non-threatened). In addition, we calculated the percentage of species correctly classified (PCC), the percentage of non-threatened species correctly classified (specificity), and the percentage of threatened species correctly classified (sensitivity).

The proportion of non-threatened species in our data was high (i.e. most species were not currently threatened according to the IUCN). To assess whether a high proportion of species were classified correctly by our models (as a result of the small percent of at-risk species), we undertook a comprehensive sensitivity analysis. We systematically resampled our data to show how the accuracy measures for threat status predictions differed among samples having different proportions of threatened to non-threatened species (see Table S1). We did all analyses using the R software [36], used the ‘rpart’ package to build classification trees and package ‘randomForest’ to build random forests [37].

To correlate extinction risk with geographic area in Brazil, we overlaid digital maps illustrating the geographic distribution of all species predicted to be threatened (or not) by our model onto an equal-area grid of the entire Brazilian territory (with spatial resolution of 0.25°x 0.25° of latitude/longitude). We created maps of four species groups according to our model predictions: (A) species predicted to be threatened, (B) predicted to be non-threatened, (C) predicted to be threatened by our model but currently listed as non-threatened according to the IUCN (latent risk species), and (D) those predicted as non-threatened by our model, but currently threatened according to the IUCN (bad luck species). After this, we created distribution maps of proportional species richness (total number of species in each group described above divided by the total number of species occurring in each grid cell).

We then overlaid digital maps of species distribution onto the network of PAs in Brazil and quantified species richness and the proportion of species within a geographic range covered by PAs (i.e. the current level of protection of species). We also evaluated the representation of species in the PAs network, considering a species as ‘represented’ when at least one grid cell of their range overlapped with PAs. We analyzed species representation considering different PAs categories: (1) only strict PAs (IUCN I-IV categories), and (2), all PAs (IUCN I-IV and VI categories).

## Results

**Figure 1:**
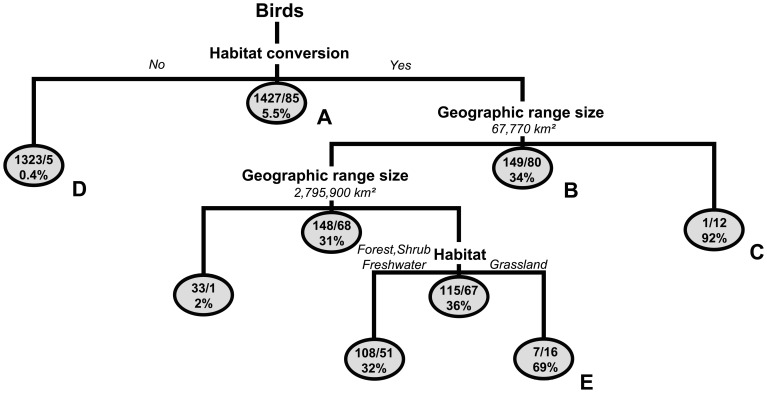
Classification tree showing bird extinction risk in Brazil (according to external threats and intrinsic biological features) and the relative risk of species. Labeled nodes are referenced in main text.

Classification trees (expanded and optimal) and random forest models both accurately predicted bird extinction risk (see summary statistics in Table 1 and Table S1). The optimal tree (Fig. 1) had four splits (pruned based on the results of 10 cross-validations, Fig. S1). Despite high model accuracy in predicting extinction risk, the model sensitivity was low. This result indicated that a model stating there are no species at risk' would be correct almost 95% of the time - a very accurate model. However, the ability of our model to predict at risk species was also low. Such low sensitivity was clearly related to the unbalanced proportion between threatened (n = 85) and non-threatened species (n = 1472). Reducing this unbalanced proportion increased sensitivity values (see Table S1), which resulted in increased model accuracy when the number of threatened and non-threatened species were similar in the analyses.

**Table 1:** Accuracy measures for predictions of threat status in birds species with current geographic range size and external threats (n = 1557 species).

	Classification Model		
Accuracy Metric	Classification Tree: expanded (n = 23)	Classification Tree: optimal (n = 4)	Random Forest
PCC	97.88%	95.82%	95.3%
Specificity	99.25%	99.45%	99.45%
Sensitivity	74.11%	32.94%	23.5%
Error rate (null error = 5.4%)	2.12%	4.18%	4.7%
Kappa (*p*-value)	0.781 (<0.0001)	0.445 (<0.0001)	0.421 (<0.0001)

The optimal classification tree showed different pathways to bird extinction in addition to threshold values for features contributing to extinction. In Brazil, *ca*. 5% of the birds included in our analyses were currently threatened with extinction (see Fig. 1, node A). Extinction risk increased or decreased depending on which features were considered and how these features interacted in the model. Species threatened by habitat conversion had a higher extinction risk (34%, node B) than those not threatened by this external threat (0.4%, node D). Furthermore, those species threatened by habitat conversion and occupying geographic range size smaller than 67,770km^2^ had the highest extinction risk (92%, node C). Species with an intermediate-sized range occupying grasslands also had a high risk of extinction risk (69%, node E).

**Figure 2:**
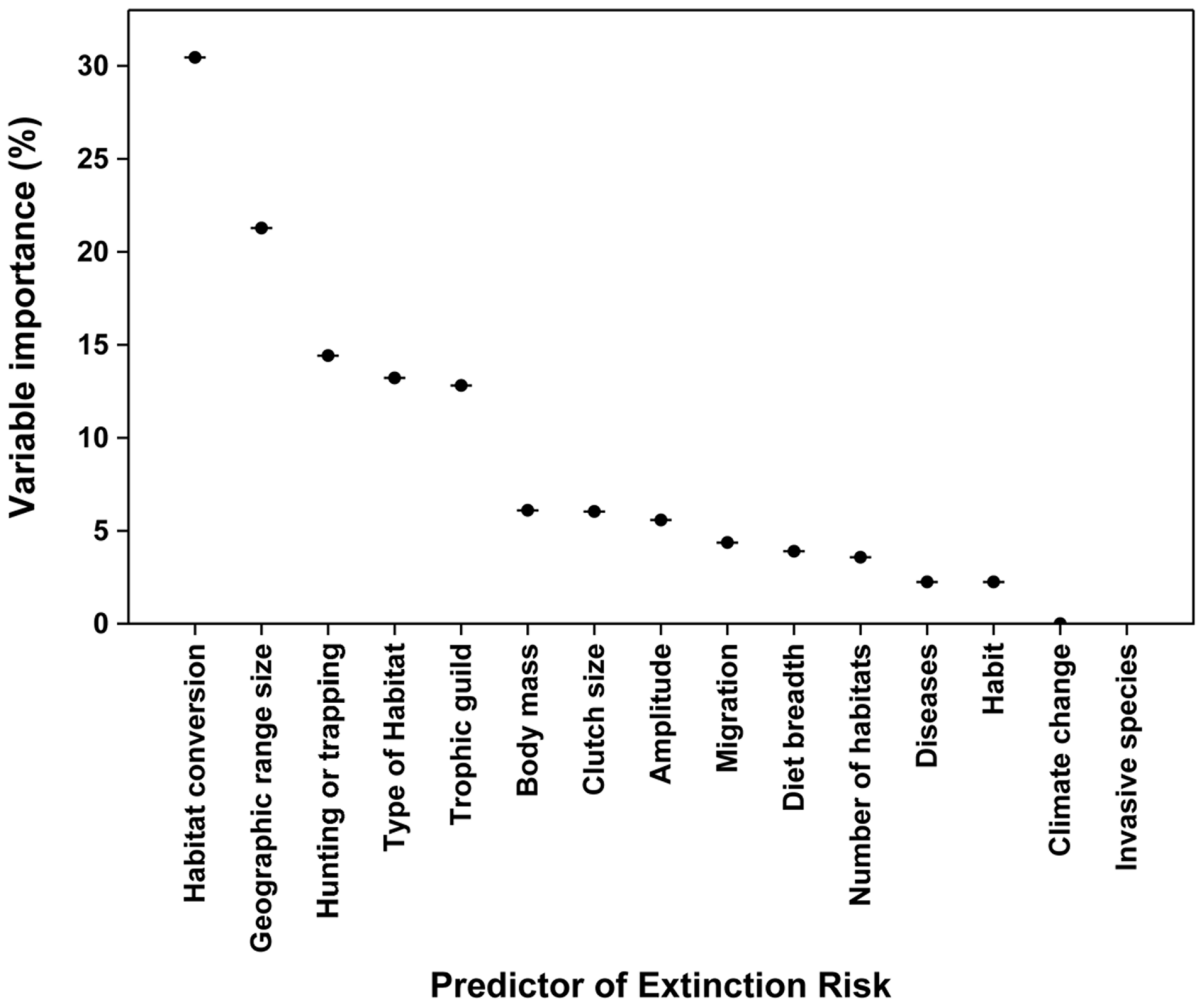
Relative importance of biological traits and external threats in predicting bird extinction risk in Brazil measured by the reduction in classification accuracy upon a stepwise removal of each trait in a set of 499 random forest trees. Error bars represent the standard deviation.

Both biological features and external threats made distinct contributions in defining extinction risk. As expected, habitat conversion was the most important feature (Fig. 2) though geographic range, hunting or trapping, type of habitat and trophic guild were also important. Even though we excluded all species from our analysis that were classified as threatened by the IUCN due to their small geographic range, this feature was still critical to defining extinction risk. In fact, one of the first major splits in the classification tree was defined by range size (small vs. large range size) (Fig.1, node B).

Our model suggest that eight species, currently classified as non-threatened by the IUCN Red List, could be threatened in the future as they share some features with threatened species, such as small range size and preferentially inhabiting grasslands: *Asthenes hudsoni*, *Alipiopsitta xanthops*, *Charitospiza eucosma*, *Cinclodes pabsti*, *Euscarthmus rufomarginatus*, *Picumnus limae, Porphyrospiza caerulescens* and *Sporophila hypochroma* (see Table S2 and Table S3).

**Figure 3:**
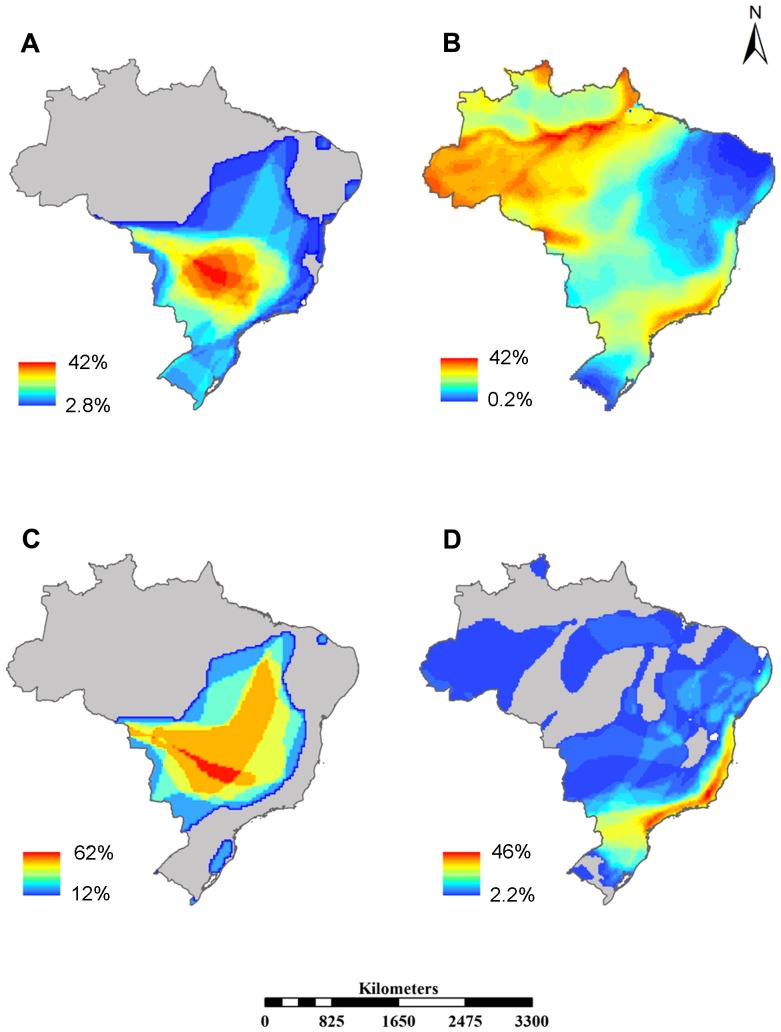
Spatial distribution of proportion of species for those predicted to be at high extinction risk (A), under low extinction risk (B), currently non-threatened but predicted by classification tree as threatened (C) and, currently threatened but predicted as non-threatened (D). The species richness is represented by graduated colors in the map.

The geographic pattern of species distribution predicted to be under high and low extinction risk were quite different. Species at high risk were concentrated mainly in the Cerrado Biodiversity Hotspot of Brazil, also an extinction risk hotspot (Fig. 3A). Species under low extinction risk were located in the northwest (Amazon Forest) and southeast (Atlantic Forest Biodiversity Hotspot) of Brazil (Fig. 3B). Species currently classified as non-threatened by the IUCN Red List, but predicted as threatened by our model (latent risk species) were also primarily located in the Cerrado, a latent risk region (Fig. 3C). On the other hand, species currently threatened, but predicted to be safe (i.e. bad luck species), concentrated mainly along the coastal line of the Brazilian Atlantic Forest (Fig. 3D).

**Figure 4:**
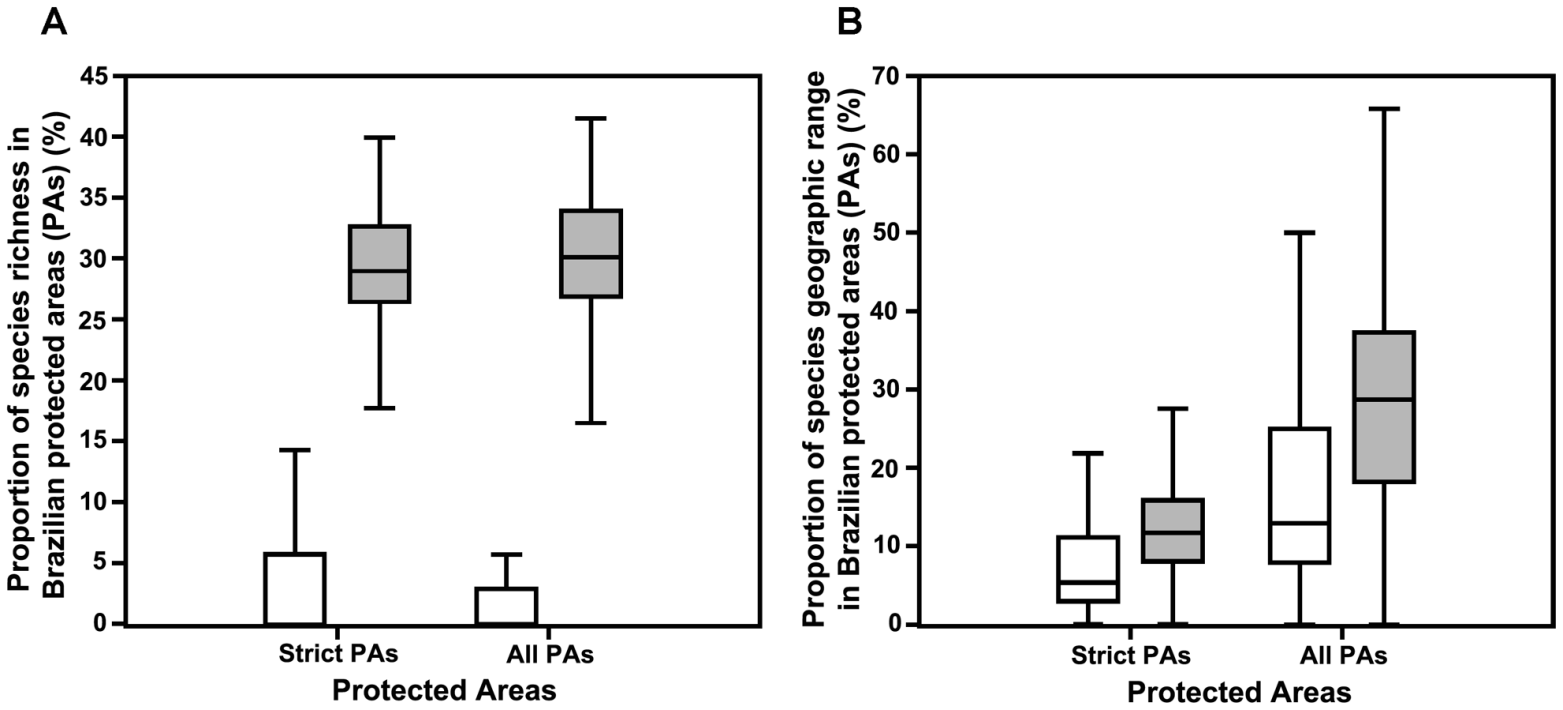
Proportion of species richness (A) and of species geographic range (B) predicted by classification tree model to be at high and low extinction risk considered protected by the current protected area network in Brazil: strict PAs (I-IV categories of IUCN) and All PAs (I-VI categories of IUCN). White bars represent species with high extinction risk, light gray represent species with low extinction risk. Species were considered as ‘protected’ when there was overlap between at least one cells grid of their range distribution and protected areas. Internal lines stand for the median. Line extensions form each box represent non-outlier range.

Most PAs sheltered close to zero percent of species predicted to be under a high extinction risk (n = 35 species protected in strict and all PAs). However, more than 30% of species under low extinction risk were represented in most PAs (Fig. 4A). Furthermore, the few species under high extinction risk whose distributions coincide with PAs have only *ca.* 5% of their range inside the strict PAs. This value increased to 10% when we also considered non-strict PAs together with strict ones. Species under low risk had *ca.* 11% of their ranges inside strict PAs and the inclusion of non-strict PAs increased their protected geographic range to almost 30% (Fig. 4B).

## Discussion

This study shows a comprehensive quantitative assessment of bird extinction risk in Brazil. We also quantified and disentangled the effect of both intrinsic biological features and external threats in defining bird extinction risk in this megadiverse country, determining the most significant factors driving bird extinctions. It is known that the effects of external threats, as well as species features, are particularly important in defining vertebrate extinction risk [2], [15], [20], [38]. The impacts of human activity, such as habitat conversion, have been largely documented as the main cause of species loss [5]. However, extinction risk predictors play a lesser or greater role defining extinction risk depending on synergic action and may only act in particular situations. For example, when species have a large range size, the effects of habitat conversion are minimized. In contrast, extinction risk changes according to the type of habitat in which a particular species lives being the highest in species occupying grasslands.

Although range size is a well-known predictor of extinction in vertebrates [15], the definition of what is considered a small or a large range-size value has previously been subjective. For birds species in Brazil, this threshold was found to be 67,000 km^2^, although previous studies with forest birds found a lower threshold of 11,000 km^2^ [20]. Therefore, thresholds may change if we only analyze particular species and its specific habitat. However, we must remember that these thresholds are still optimistic and may be overestimated. Even considering habitat conversion as an external factor, the range size we used was based on species extent of occurrences, which are often inaccurate, and by including all regions where species could occur, may overestimate the range size by dismissing unoccupied portions of remaining habitats [39]. Additionally, we should bear in mind that geographic range and bird taxonomy are still not completely understood (especially for tropical species) which could further bias our results. Amazonian birds, for example, inhabit regions with large, under-sampled areas, and their known ranges routinely expanding as new expeditions are conducted [39].

Larger body mass is also a well-known feature associated with high extinction risk in vertebrates [1], [2], [12], [16]. Species sharing similar values for this trait may be more prone to extinction due to their low abundance, slower life history, lower reproductive rates, and larger home-ranges [15], [16]. It is still important to note that some biological features are indirectly considered in each IUCN criterion used to classify species. Although not all features were necessarily considered when listing species, they could be confounded with criterions. For example, criterion A deals with population size, which is obviously related to body size, type of preferred habitat and clutch size (those three features are not formally considered in criterion A). Therefore, even after excluding species listed under criterion B, other criteria might have been confounded with the species biological features.

Grassland species had a higher extinction risk in our model. Grasslands are among the most endangered ecosystems in the world [40] and many species of grassland birds are area-sensitive [41]. This means that grassland birds with smaller range sizes would have more specific habitat requirements; consequently, they are more likely to be sensitive to habitat modifications. Surprisingly, clutch size and migration behavior did not figure significantly into our model. Long-distance migrants may be particularly vulnerable to alterations in habitat or climatic conditions in their breeding range [42]. Fecundity (measured by clutch size) is also associated with extinction risk [1], [2] given that species with low fecundity take more time to recover their population after stochastic events [43]. The same happened with habitat breadth, although this feature is intuitively associated with extinction: the more habitats a species might occupy, the higher the chance of survival given their higher environmental plasticity. The low contribution of these variables to our model could be due to data refining. With respect to habitat type we used coarser habitat categories that may not have been as sensible as specific microhabitats.

Climate change is also recognized as a threat to biodiversity [7], [44], but its impact on bird biodiversity is still poorly studied/understood in tropical regions (but see Anciães & Peterson [45] and Marini et al. [46]). However, negative effects are predicted in the future [7], [47], which in turn could be reflected in its lower importance in defining extinction risk in our model [4].

Our model is an alternative tool to predict extinction risk, even for poorly known species for which we have little information about population dynamics [15]. Predictive models may help in prioritizing species for conservation action, rather than by using their current threat status [17]. Highlighting species predicted to have a high extinction risk, but currently listed as non-threatened, is of particular importance [19] given that these species usually attract minimal conservation attention [11].

This study indicated that some species currently classified as non-threatened by the IUCN are predicted as having a threatened status in our models. However, most of these species have already been recognized by the IUCN as having an elevated risk of extinction, and are currently classified as Near-Threatened species (Table S2). Nevertheless, we believe resources could be more efficiently allocated applied to the conservation of these species and avoiding forthcoming extinctions [11]. Moreover, extinction risk models are critical tools for evaluating species defined under IUCN criterion E, which accounts for quantitative analysis. Quantitative analysis which estimates the extinction probability of a taxon based on known life history, habitat requirements, threats and any specified management options. To our knowledge this study is the first to report a comprehensive quantitative analysis on the extinction risk of bird species in Brazil.

However, the accuracy of our models was clearly dependent on the ratio between threatened and non-threatened species. Our models had a high predictive accuracy for detecting species that were not at risk, but our ability to predict species at risk only increased when the number of threatened and non-threatened species were similar (see Table S1). Previous works also show a similarly low sensitivity in detecting threatened species [15], [42]. This is an important consideration for conservation efforts, as the ability to predict for at-risk species is critical. Though our models could foster discussion or be used to evaluate the inclusion of species under criterion E, they should be applied with prudence given their inherent statistical limitations.

Extinction risk is influenced by external factors [2], [17]. Even species that are relatively safe when only intrinsic features are considered may be severely affected by external threats (such as habitat conversion), increasing their probability of extinction. In addition, threats to biodiversity vary across species and sites. Our results corroborated previous studies assessing the pattern of geographic threat in South America that indicate the Cerrado region and Atlantic Forest as the main regions sheltering species at a higher risk[42], [48]–[51]. Jenkins & Pimm [52] argued that sites of high conservation priority are also those which contain threatened habitats, indicating a link between habitat loss and threatened birds in the Atlantic forest region. The Cerrado region and Atlantic Forest are under higher anthropogenic pressure in Brazil [50], [53]–[55], and in the case of Atlantic forest, harbor species with the smallest range size [49]. On the other hand, the Amazon forest is largely undisturbed and species at low extinction risk do not share geographic range with large human populations, unlike those of the Atlantic Forest ([17], but see Luck [56]).

Nevertheless, given the fast growth rate of the human population, it would be better to identify the latent areas at risk and proactively set priorities for the conservation biodiversity [17], [57], [58]. We indicated that the Cerrado and Atlantic forest regions harbor species of concern. The Cerrado region is a highly heterogeneous domain, consisting primarily of seasonal tropical savanna that has been enormously and rapidly transformed by human activities with a concomitant los in species [51], [55]. Furthermore, only 2.2% of this area is currently under legal protection [55], [59]. Although grassland habitats have a high biodiversity value, they are under jeopardy, barely protected by currently PAs, have inadequate management [60], [61], habitat conversion is occurring rapidly [55] and their bird populations are in decline [51].

The network of PAs in Brazil must be broadened to guarantee the inclusion of more species, given that many species are poorly represented in the majority of PAs. Clearly, current PAs are not adequate to protect species having high extinction risk. Similar to the global protected-area system [62], the Brazilian system is far from complete [63], [64]. A recurrent and unresolved issue in conservation biology is about how much of a biological element’s distribution actually needs to be represented in conservation areas [57], despite efforts to establish a blanket target [62]. Even without such a target, including intrinsic biological features in conservation planning analyses seems key to producing more effective and ecologically sound conservation plans [21], [65], [66]. Additionally, despite increased species representation with the inclusion of non-strict PAs, various types of economic activities happening within them could threat more sensitive species.

An increasing knowledge about species will probably serve to refine extinction risk models. Despite academic efforts to find better spatial solutions that account for biological features [21], the solution are poorly applied during the selection of proposed conservation areas. We hope the results of this study will help to highlight the importance of natural history and ecology in conservation biology and spatial conservation planning.

## Supporting Information

Figure S1 **Relative error for the classification tree determined by 10 cross-validations.** The optimal tree is indicated by a filled circle and has eight splits.
(TIF)

Table S1 **Accuracy measures for predictions of threat status for different samples of species (The** ***n*** **values showed represent all species analyzed: 85 threatened species, plus random samples of non-threatened bird species).**
(DOC)

Table S2 **Species currently classified as non-threatened by IUCN** [**1**] **but predicted as threatened by our classification tree model (optimal tree).** NT: Near threatened, LC: Least Concern. The column “Classification tree node” show the species position in the Classification tree optimal (see Fig. 1).
(DOC)

Table S3 **Species assessed in this study along with their current IUCN Red List status, population trend, and predicted extinction risk accordingly to our model.**
(XLS)
