# Supplementary figures and images for: A Comprehensive Quantitative Assessment of Bird Extinction Risk in Brazil
Source: PLoS One. 2013 Aug 12;8(8):e72283. doi: 10.1371/journal.pone.0072283 (PMC3741389; doi:10.1371/journal.pone.0072283)

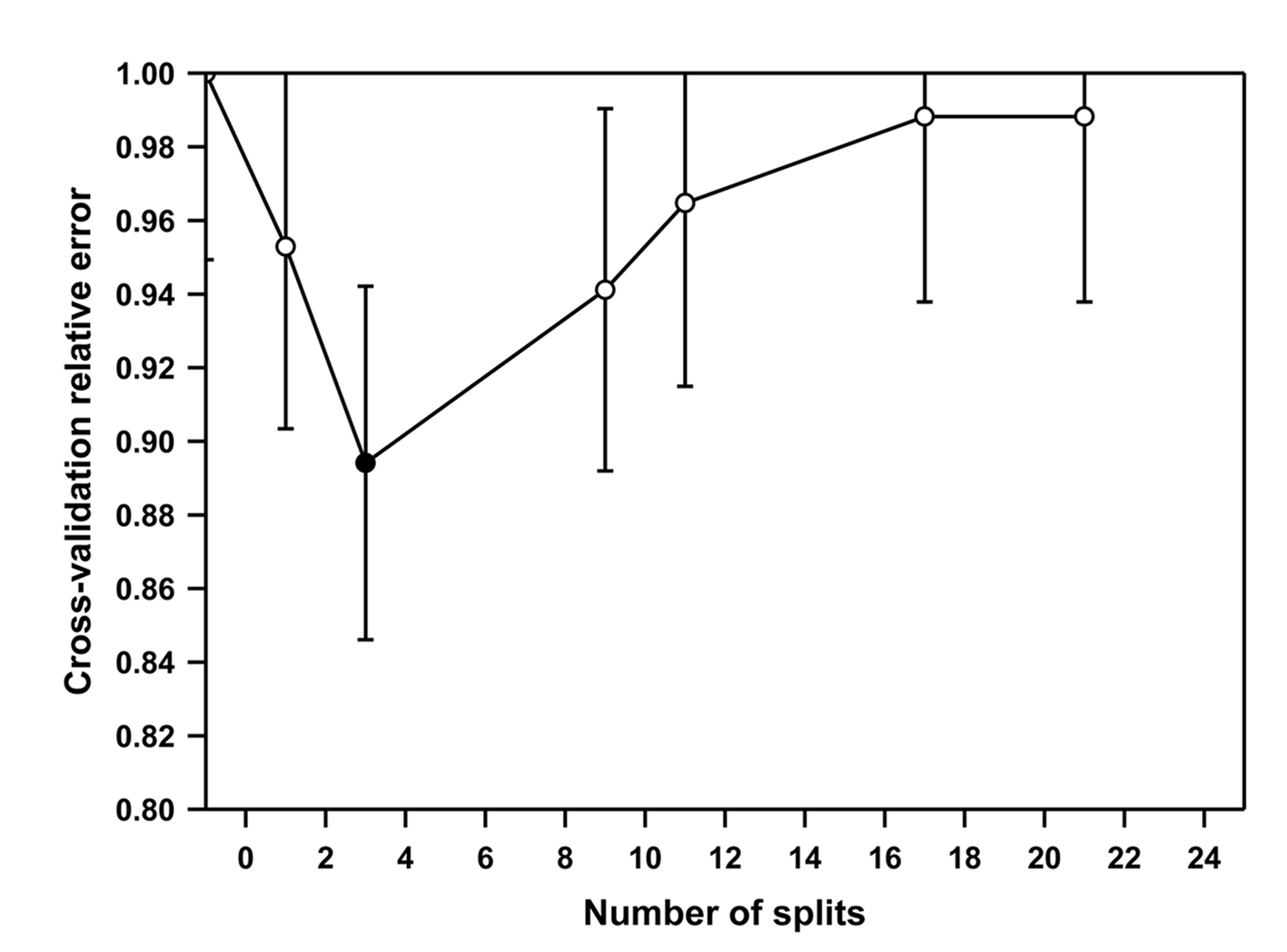

Supplement: Figure S1 — Relative error for the classification tree determined by 10 cross-validations. The optimal tree is indicated by a filled circle and has eight splits. (TIF) [file pone.0072283.s001.tif]
