# Supplementary material for: A Comprehensive Quantitative Assessment of Bird Extinction Risk in Brazil
Source: PLoS One. 2013 Aug 12;8(8):e72283. doi: 10.1371/journal.pone.0072283 (PMC3741389; doi:10.1371/journal.pone.0072283)
Supplement: Table S1 — Accuracy measures for predictions of threat status for different samples of species (The n values showed represent all species analyzed: 85 threatened species, plus random samples of non-threatened bird species). (DOC) [file pone.0072283.s002.doc]

**Supporting Information**

Table S1: Accuracy measures for predictions of threat status for different samples of species (The *n* values showed represent all species analysed: 85 threatened species, plus random samples of non-threatened bird species).

|  |  |  |  |  |  |  |  |  |  |  |  |  |  |
| --- | --- | --- | --- | --- | --- | --- | --- | --- | --- | --- | --- | --- | --- |
| n | Threat/No-threat Rate |  | Classification Tree expanded | | |  | Classification Tree prunned | | |  | Random Forest | | |
|  |  |  | PCC | Specificity | Sensitivity |  | PCC | Specificity | Sensitivity |  | PCC | Specificity | Sensitivity |
| n=170 | 1/1 |  | 92.0 | 89.7 | 94.3 |  | 89.0 | 82.8 | 95.3 |  | 86.1 | 85.1 | 87.2 |
| n=255 | 1/2 |  | 93.8 | 95.4 | 90.7 |  | 81.5 | 76.4 | 91.9 |  | 76.5 | 84.5 | 60.5 |
| n=340 | 1/3 |  | 95.4 | 98.8 | 84.9 |  | 95.4 | 98.8 | 84.9 |  | 81.7 | 93.8 | 49.0 |
| n= 425 | 1/4 |  | 94.9 | 97.7 | 83.7 |  | 95.2 | 98.0 | 83.7 |  | 76.5 | 84.5 | 60.5 |
| n=510 | 1/5 |  | 92.3 | 97.7 | 65.1 |  | 92.3 | 97.7 | 65.1 |  | 87.3 | 96.8 | 39.5 |
| n= 595 | 1/6 |  | 95.5 | 98.5 | 78.2 |  | 91.6 | 98.3 | 52.8 |  | 88.1 | 96.9 | 34.9 |
| n=680 | 1/7 |  | 95.4 | 98.7 | 68.6 |  | 92.7 | 99.7 | 36.0 |  | 92.1 | 99.1 | 34.9 |
| n =765 | 1/8 |  | 96.1 | 99.6 | 64.0 |  | 93.2 | 99.6 | 34.9 |  | 91.5 | 98.7 | 25.6 |
| n= 850 | 1/9 |  | 95.4 | 99.7 | 52.3 |  | 93.5 | 99.2 | 36.0 |  | 92.2 | 98.5 | 27.9 |
| n =935 | 1/10 |  | 95.8 | 99.5 | 51.2 |  | 96.1 | 99.5 | 55.2 |  | 93.5 | 99.0 | 26.7 |
| n=1360 | 1/15 |  | 96.6 | 99.8 | 53.5 |  | 94.9 | 99.5 | 34.9 |  | 93.8 | 99.2 | 23.3 |
|  |  |  |  |  |  |  |  |  |  |  |  |  |  |

Table S3: Species currently classified as non-threatened by IUCN [28] but predicted as threatened by our classification tree model (optimal tree). NT: Near threatened, LC: Least Concern. The column “Classification tree node” show the species position in the Classification tree optimal (see Fig. 1).

| **Species** | **Common Name** | **IUCN Red List status** | **Population trend (IUCN)** | **Year assessed** | **Classification tree node** |
| --- | --- | --- | --- | --- | --- |
| *Asthenes hudsoni* (Sclater, 1874) | Hudson's Canastero | NT | decreasing | 2009 | Node D |
| *Cercibis oxycerca* (Spix, 1825) | Sharp-tailed Ibis | LC | - | 2009 | Node D |
| *Cinclodes pabsti* Sick, 1969 | Long-tailed Cinclodes | NT | decreasing | 2008 | Node D |
| *Ortalis superciliaris* Gray, 1867 | Buff-browed Chachalaca | NT | decreasing | 2008 | Node E |
| *Penelope pileata* Wagler, 1830 | White-crested Guan | NT | decreasing | 2008 | Node E |
| *Picumnus limae* Snethlage, 1924 | Ochraceous Piculet | LC | - | 2010 | Node B |
| *Poecilotriccus albifacies* (Blake, 1959) | White-cheeked Tody-flycatcher | LC | - | 2009 | Node B |
