# Supplementary material for: A Comprehensive Quantitative Assessment of Bird Extinction Risk in Brazil
Source: PLoS One. 2013 Aug 12;8(8):e72283. doi: 10.1371/journal.pone.0072283 (PMC3741389; doi:10.1371/journal.pone.0072283)
Supplement: Table S2 — Species currently classified as non-threatened by IUCN [1] but predicted as threatened by our classification tree model (optimal tree). NT: Near threatened, LC: Least Concern. The column “Classification tree node” show the species position in the Classification tree optimal (see Fig. 1). (DOC) [file pone.0072283.s003.doc]

**Supporting Information**

Table S2 Species currently classified as non-threatened by IUCN [1] but predicted as threatened by our classification tree model (optimal tree). NT: Near threatened, LC: Least Concern. The column “Classification tree node” show the species position in the Classification tree optimal (see Fig. 1).

| **Species** | **Common Name** | **IUCN Red List status** | **Population trend (IUCN)** | **Year assessed** | **Extintion Risk** | **Classification tree node** |
| --- | --- | --- | --- | --- | --- | --- |
| *Asthenes hudsoni* (Sclater, 1874) | Hudson's Canastero | NT | decreasing | 2009 | 69% | E |
| *Alipiopsitta xanthops* (Spix, 1824) | Yellow-faced Amazon | NT | decreasing | 2008 | 69% | E |
| *Charitospiza eucosma* Oberholser, 1905 | Coal-crested Finch | NT | decreasing | 2008 | 69% | E |
| *Cinclodes pabsti* Sick, 1969 | Long-tailed Cinclodes | NT | decreasing | 2008 | 69% | E |
| *Euscarthmus rufomarginatus* (Pelzeln, 1868) | Rufous-sided Pygmy | NT | decreasing | 2008 | 69% | E |
| *Picumnus limae* Snethlage, 1924 | Ochraceous Piculet | LC | - | 2010 | 92% | C |
| *Porphyrospiza caerulescens* (Wied, 1830) | Blue Finch | NT | decreasing | 2008 | 69% | E |
| *Sporophila hypochroma* Todd, 1915 | Rufous-rumped Seedeater | NT | decreasing | 2008 | 69% | E |
|  |  |  |  |  |  |  |
